# Fused filament fabrication of radiopaque barium sulfate-reinforced polyetheretherketone composites for thin-walled patient-specific orbital reconstruction

**DOI:** 10.1007/s10856-026-07111-5

**Published:** 2026-07-25

**Authors:** Jokin Zubizarreta Oteiza, Yannick Krieger, Daniel Seiler, Philippe C. Cattin, Florian M. Thieringer, Neha Sharma

**Affiliations:** 1 Clinic of Oral and Cranio-Maxillofacial Surgery and 3D Print Lab, University Hospital Basel, Basel, Switzerland; 2 Medical Additive Manufacturing Research Group (Swiss MAM), Department of Biomedical Engineering, University of Basel, Allschwil, Switzerland; 3 3DSystems, Munich, Germany; 4 Institute for Medical Engineering and Medical Informatics, University of Applied Sciences and Arts Northwestern Switzerland, Muttenz, Switzerland; 5 Center for Medical Image Analysis & Navigation (CIAN), Department of Biomedical Engineering, University of Basel, Allschwil, Switzerland; 6 Duke-NUS Medical School, Singapore, Singapore

## Abstract

Fused filament fabrication (FFF) of high-performance thermoplastic composites for medical applications remains challenging due to the need to meet geometric precision, mechanical, and functional requirements. This study presents the first systematic evaluation of FFF radiopaque barium sulfate-reinforced polyetheretherketone (BaSO₄-PEEK) for patient-specific orbital reconstruction, addressing dimensional accuracy, mechanical properties, and surgical precision in thin-walled implants. Six implants per material group (unfilled PEEK and 20 wt% BaSO₄-PEEK) with 0.8 mm nominal wall thickness and complex orbital geometries were fabricated and evaluated. Optical scanning showed submillimeter-level dimensional accuracy, with root-mean-square error (RMSE) of 0.31 ± 0.09 mm for unfilled PEEK and 0.33 ± 0.08 mm for BaSO₄-PEEK, confirming that incorporating an organic filler does not compromise manufacturability. Biomechanical testing revealed that FFF BaSO₄-PEEK implants maintained adequate puncture strength (17.58 ± 0.46 N/mm²), comparable to titanium mesh. Radiographically, BaSO₄-PEEK implants provided a 38% higher contrast-to-noise ratio than titanium and eliminated metal artefacts that obscured adjacent soft tissues. Cadaveric implantation demonstrated reconstruction accuracy (RMSE) of 0.61 ± 0.15 mm, which was better than that of manually contoured titanium mesh. These results establish FFF as a viable manufacturing method for complex, thin-walled, radiopaque PEEK composite orbital implants that provide sufficient dimensional control and mechanical performance in clinical scenarios.

**Figure:**
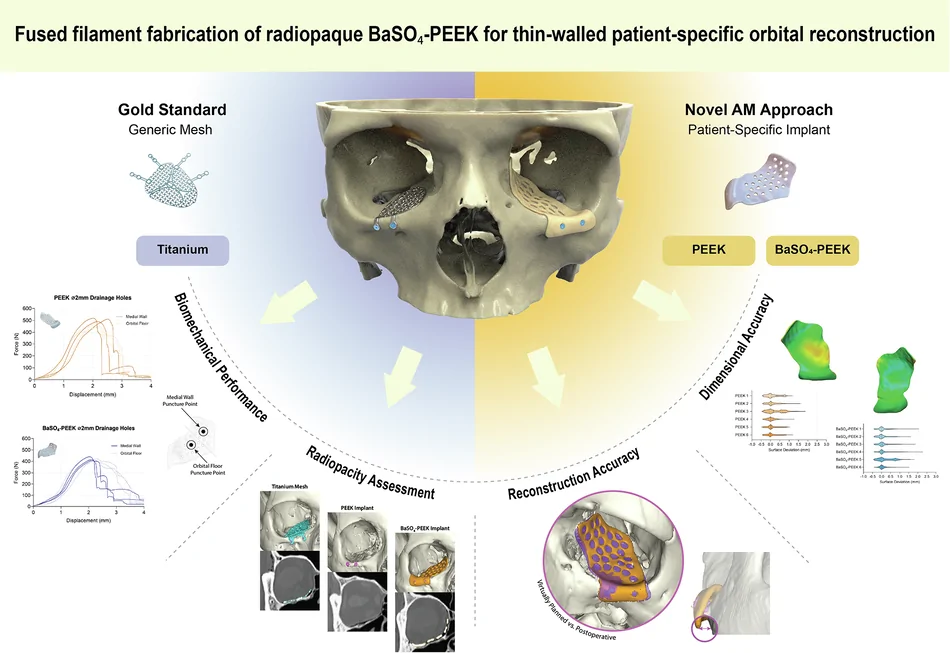

## Highlights

- Fused filament fabrication (FFF)-fabricated BaSO₄-PEEK orbital implants achieved submillimeter dimensional accuracy (RMSE 0.33 ± 0.08 mm) with 0.8 mm wall thickness.
- Radiopaque FFF PEEK provided a 38% higher contrast-to-noise ratio than titanium while eliminating metal artefacts.
- Puncture strength of radiopaque FFF PEEK (17.58 ± 0.46 N/mm²) exceeded physiological requirements with substantial safety margins.
- Cadaveric validation confirmed reconstruction accuracy of 0.61 ± 0.15 mm RMSE for radiopaque FFF PEEK patient-specific orbital implants.

## Introduction

High-performance thermoplastic composites enable geometric customisation in precision medical devices through advanced additive manufacturing (AM) technologies, combining material functionality with dimensional accuracy that is unattainable with traditional fabrication methods [1, 2]. Fused filament fabrication (FFF), a filament-based material extrusion technology, of polyetheretherketone (PEEK) and its composites has attracted particular interest for craniofacial reconstruction because of PEEK’s bone-like elastic modulus (3–4 GPa), biocompatibility, and artefact-free medical imaging characteristics compared with metallic implants [3, 4]. However, the radiolucency of unfilled PEEK fundamentally limits postoperative radiographic evaluation, necessitating radiopaque formulations that maintain processability while providing clinical visibility [5, 6].

Thin-walled orbital implants (<1 mm) pose demanding requirements due to complex three-dimensional (3D) geometries that conform to anatomical contours, submillimeter dimensional accuracy for precise fit, and adequate mechanical performance under physiological loading [7, 8]. Current clinical approaches rely on manually contoured titanium mesh, which provides radiographic visibility but generates severe artefacts on computed tomography (CT) and magnetic resonance imaging (MRI) that obscure adjacent soft tissues, while manual adaptation of these meshes introduces operator-dependent geometric variability that compromises anatomical precision [9–11]. Additional challenges include implant–tissue adhesions that may progress to orbital adherence syndrome, resulting in restricted ocular motility and eyelid retraction, as well as the inherent limitations of manual intraoperative contouring, which reduce anatomical precision in complex defects [12–14]. Sharp edges from manually adapted meshes can lacerate orbital fat and soft tissues, while material stiffness may cause severe postoperative deformation threatening adjacent structures [15–18]. These limitations motivate the development of patient-specific polymer composite alternatives that combine precise geometric 3D volumetric reconstruction, radiographic functionality, and artefact-free imaging.

The incorporation of barium sulfate (BaSO₄) as a radiopaque filler in PEEK matrices offers a potential solution, with concentrations of 20–30 wt% providing radiographic visibility without generating metal artefacts [19, 20]. However, the incorporation of inorganic fillers fundamentally alters polymer melt rheology and crystallisation kinetics, potentially compromising the dimensional accuracy and mechanical properties essential for thin-walled medical device applications [21]. The rheological challenges include increased melt viscosity, altered flow behaviour during printing, and intra- and interlayer bonding characteristics that can affect layer-by-layer build quality in FFF 3D printing [22]. Optimising filler concentration requires balancing radiographic performance, processability constraints, and mechanical performance, as excessive loading can critically compromise material flow and structural integrity.

Despite the clinical promise of radiopaque PEEK composites, systematic characterisation of FFF printability for BaSO₄-PEEK in thin-walled geometric applications remains limited. Previous studies with unfilled PEEK report dimensional accuracies of 0.3–0.8 mm root-mean-square error (RMSE) for thicker-walled medical devices (cranial implants >3.5 mm) [23, 24], but the impact of inorganic fillers on manufacturing precision in submillimeter wall structures has not been quantified. The fundamental challenge lies in achieving submillimeter dimensions despite rheological changes induced by filler incorporation, such as increased viscosity, altered crystallisation, and altered interlayer fusion, while maintaining adequate mechanical performance and radiographic functionality. Whether material optimisation and processing parameter adaptation can overcome these competing constraints to enable functional radiopaque composites in demanding thin-walled applications remains experimentally unvalidated. Furthermore, comprehensive functional validation that combines manufacturing accuracy, mechanical performance, and surgical placement accuracy for FFF-fabricated radiopaque PEEK has not been reported previously.

This study provides the first systematic functional validation of radiopaque BaSO₄-PEEK composites, fabricated specifically via FFF, for thin-walled patient-specific orbital reconstruction, through four domains: (1) dimensional accuracy quantification via optical metrology to assess manufacturing precision; (2) mechanical performance evaluation under physiologically relevant puncture loading; (3) radiographic functionality assessment quantifying radiopacity and artefact generation relative to titanium mesh; and (4) surgical reconstruction accuracy validation through cadaveric implantation. Together, these analyses establish whether FFF-fabricated radiopaque PEEK can meet the requirements for point-of-care (POC) manufacturing of patient-specific orbital implants, addressing key limitations of current titanium mesh solutions.

## Materials and methods

### Implant design and fabrication

High-resolution CT imaging data from nine anonymised cadaveric specimens were acquired (SOMATOM go.Now, Siemens Healthineers AG, Forchheim, Germany) at the International Bone Research Institute (IBRA). Digital Imaging and Communications in Medicine (DICOM) datasets with 0.8 mm slice thickness were imported into medical image processing software (Materialise Mimics Innovation Suite v. 26.0, Materialise NV, Leuven, Belgium), where 3D volumetric reconstructions of orbital anatomy were generated through semi-automated segmentation based on bone-specific Hounsfield Units (HU) with manual refinement. The reconstructed models were exported as Standard Tessellation Language (STL) files for further processing. Anatomical biomodels were fabricated for each specimen using a desktop FFF 3D printer (Bambu Lab X1 Carbon, Bambu Lab, Shenzhen, China) with polylactic acid (PLA) filament (Matte Ivory White, Bambu Lab, Shenzhen, China) and used for patient-specific implant (PSI) fit verification and titanium mesh pre-contouring.

Orbital implants were fabricated across three material groups (*n* = 6 per group): titanium mesh, unfilled PEEK PSI, and BaSO₄-PEEK PSI. Implant design was stratified by fracture complexity to reflect appropriate clinical applications for each material [25]. Pre-fabricated stock titanium orbital meshes (Medartis Orbital Plating System OPS 1.5, M-4440, 0.3 mm thickness, Medartis AG, Basel, Switzerland) were designated for simpler orbital fractures involving the orbital floor and/or medial wall in anterior and middle orbital regions (zones 1–2), representing typical indications for pre-contoured titanium meshes in clinical practice.

Patient-specific PEEK implants were digitally modelled for complex defects involving the entire orbital floor and medial wall, extending into the posterior third (zone 3), with a missing bony ledge, representing a clinical scenario where patient-specific implants offer the most significant advantage over manually contoured meshes due to geometric complexity and the lack of posterior anatomical landmarks. Design was performed using computer-aided design (CAD) software (Geomagic Freeform Plus v. 2022.0.34, Hexagon AB, Stockholm, Sweden) with orbital anatomy segmented from CT data, including the infraorbital rim, posterior orbital floor ledge, maxillary sinus superior wall, ethmoid air cells, and optic canal. For each specimen, implant geometry was individually optimised with screw hole positions placed in regions with adequate bone stock. Finalised implant designs were exported as STL files for fabrication.

Two commercially available medical-grade (long-term, implantable) PEEK formulations were used: unfilled PEEK (Vestakeep® i4 3DF, Evonik Industries AG, Essen, Germany) and radiopaque BaSO₄-PEEK incorporating 20 wt% barium sulfate (Vestakeep® iC4520 3DF, Evonik Industries AG, Essen, Germany). Each PEEK orbital implant was test-fitted onto its corresponding 3D-printed anatomical biomodel to verify anatomical conformity and screw hole alignment.

All PEEK orbital implants were manufactured using a high-temperature FFF 3D printer (EXT 220 MED, formerly Kumovis R1, 3D Systems, Rock Hill, South Carolina, USA) with printing parameters detailed in Table 1.

**Table 1:** Fused filament fabrication (FFF) printing parameters for pure polyetheretherketone (PEEK) and radiopaque BaSO_4_-PEEK orbital implants

Parameter	Value
Nozzle temperature	420–430 °C
Nozzle diameter	0.4 mm
Chamber temperature	230 °C
Build platform temperature	250 °C
Layer height	0.26 mm
Perimeter shells	1
Print speed	1000 mm/min
Extrusion multiplier	0.96

The printing parameters were established in accordance with manufacturer recommendations for medical-grade PEEK formulations and validated through preliminary fabrication trials (*n* = 3 per material). The nozzle temperature range (420–430 °C) was selected to ensure complete polymer melting while minimising thermal degradation, with BaSO₄-PEEK requiring the upper end of the range (430 °C) to accommodate the increased melt viscosity associated with inorganic filler incorporation. The build platform temperature (250 °C) was maintained above the PEEK glass transition temperature (~143 °C) to reduce thermal gradients during layer deposition. Crystallinity was controlled by maintaining the heated build chamber at 230 °C, ensuring consistent crystallisation kinetics across layers. Warping was mitigated by combining a heated chamber, a heated build platform (250 °C), and optimised support structures. Layer height (0.26 mm) was balanced with manufacturing resolution and build-time efficiency, while print speed (1000 mm/min) was optimised to ensure adequate interlayer bonding without inducing flow instabilities in the composite melt.

**Fig. 1:**
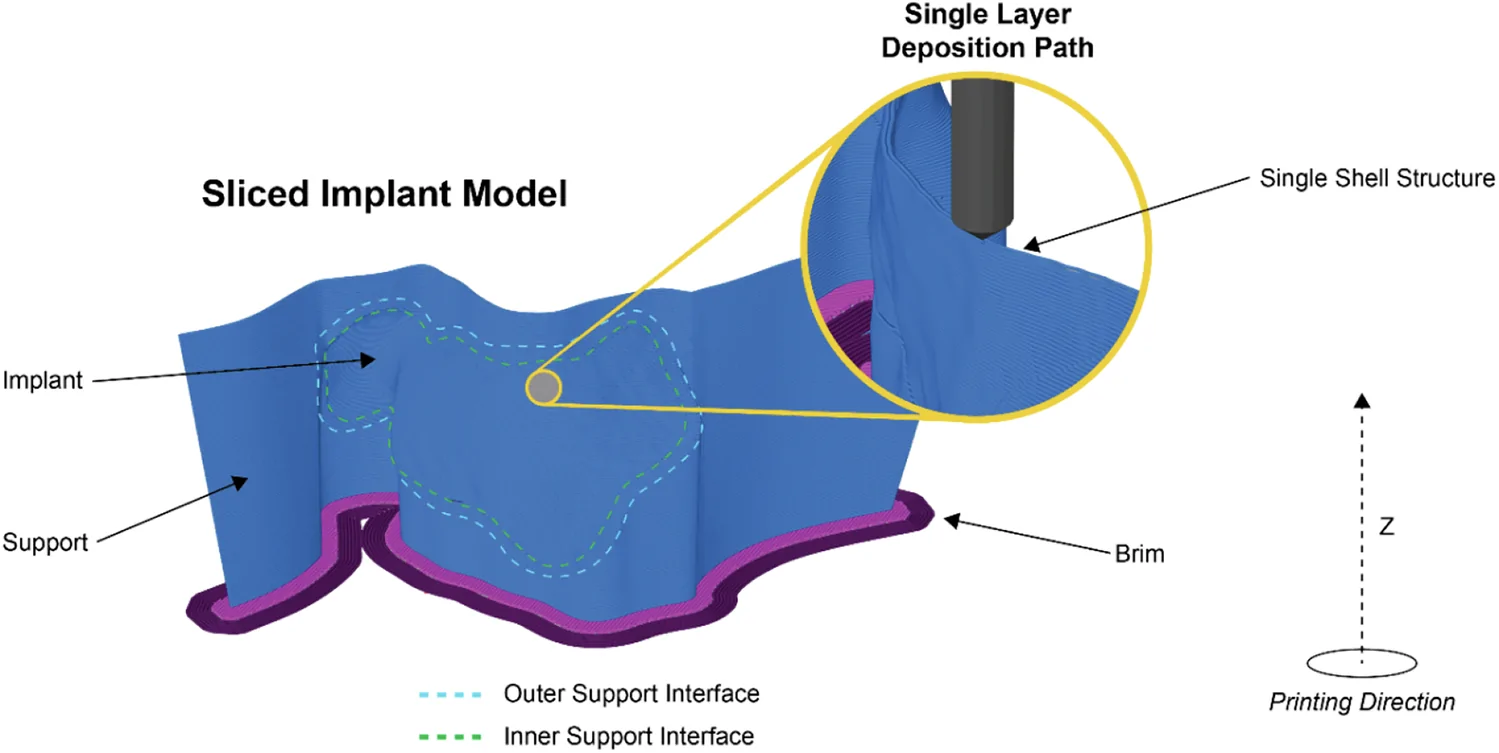
Sliced model of a Fused Filament Fabrication (FFF) patient-specific orbital implant showing build orientation and support configuration. The implant (blue) is oriented with the orbital floor surface perpendicular to the build platform, surrounded by extended support structures and a brim for build-plate adhesion. Outer (light blue dashed) and inner (green dashed) support interfaces define the contact regions between the implant and support structure. The magnified detail view illustrates the single-layer deposition path, revealing the single-shell structure of the thin-walled implant. The Z-axis indicates the printing (build) direction

All implants were fabricated with a consistent build orientation: the orbital floor surface was perpendicular to the build platform (90° relative to the orbital floor plane) to minimise support-structure contact with anatomically critical surfaces, and layer interfaces were aligned perpendicular to anticipated physiological loading directions. Owing to the 0.8 mm wall thickness, the print path consisted of perimeter shell contours (two contour paths per layer at a 0.4 mm nozzle diameter) without internal infill. Extended support structures surrounding each implant were designed and incorporated during fabrication to ensure printability of thin-walled geometries, with a 0.2 mm support-to-part interface gap to facilitate removal while maintaining geometric accuracy (Simplify3D v4.1.2, Cincinnati, Ohio, USA). A representative slicer view showing build orientation, support structure, and the single-shell layer structure is provided in Fig. 1. The build chamber atmosphere was maintained throughout fabrication to minimise thermal oxidation at elevated processing temperatures.

Manufacturing success was defined as completion without print failures (nozzle clogging, layer delamination, or warping >2 mm) and post-processing without structural damage. All 12 PEEK implants (6 unfilled PEEK, 6 BaSO₄-PEEK) achieved 100% manufacturing success, demonstrating process robustness across both formulations.

Following fabrication, implants underwent standardised post-processing performed by a single trained operator to minimise inter-operator variability: (1) support structure removal using precision micro-cutters and abrasive instruments; (2) edge refinement with progressive grit sandpaper (400–1200 grit) to eliminate support interface artefacts; (3) fixation hole preparation via 1.3 mm pilot drilling followed by 1.5 mm finishing (3000 rpm) to accommodate fixation screws; (4) drainage hole creation at 2 mm or 3 mm diameter as specified per design. For each PEEK formulation, three implants with 2 mm drainage holes and three with 3 mm drainage holes were fabricated to assess the effect of feature size on mechanical properties, thereby representing clinically relevant configurations.

### Dimensional accuracy assessment

The geometric fidelity of FFF 3D-printed implants was evaluated by comparing as-printed physical implants to their corresponding digital design models. Implant thickness was measured at three predefined anatomical locations (infraorbital rim, orbital floor, and medial wall) using a digital micrometre (DML DM1025, Digital Micrometres Ltd, Sheffield, United Kingdom).

Each implant was then digitised using a high-resolution structured-light optical scanner (3Shape E4, 3Shape A/S, Copenhagen, Denmark), and the resulting point cloud was processed to create triangulated surface meshes. Scanned implant meshes were imported into design-analysis software (Materialise 3-matic v. 18, Materialise NV, Leuven, Belgium) for dimensional comparison with original CAD models. Best-fit alignment was performed using iterative closest point (ICP) algorithms to minimise spatial discrepancies between surfaces. Dimensional accuracy was quantified using the root-mean-square error (RMSE), defined as:

$${\rm{RMSE}}=\sqrt{\frac{1}{{\rm{n}}}\mathop{\sum }\limits_{{\rm{i}}=1}^{{\rm{n}}}{\left({{\rm{d}}}_{{\rm{i}}}\right)}^{2}}$$

where *d*_*i*_ denotes the point-to-surface deviation for each corresponding vertex across the aligned meshes. Lower RMSE values indicate higher geometric fidelity of the 3D-printed implant relative to the digital design. In addition, colour-mapped 3D deviation plots were generated to support qualitative assessment of local and global surface deviations.

### Biomechanical testing

Titanium mesh was included as a biomechanical reference reflecting its current role as the clinical standard for orbital reconstruction, widely used for both simple and complex defects when patient-specific solutions are unavailable. Testing protocols were selected to evaluate clinically relevant performance metrics for patient-specific orbital implants. Puncture resistance was prioritised over tensile testing because orbital implants primarily experience localised pressure loading from trauma and surgical manipulation rather than uniaxial tension. The curved, thin-walled geometry (0.8 mm) precludes standard tensile specimen preparation, and puncture testing directly simulates the clinical failure mode relevant to this application, following standard practice for medical device proof-of-concept studies, with primary validation provided by cadaveric surgical assessment.

Biomechanical characterisation of FFF 3D-printed patient-specific orbital implants was performed to assess their functional performance under physiological loading conditions. Six implants from each PEEK material group (unfilled PEEK and BaSO₄-PEEK) were evaluated, with two measurement points per implant (orbital floor and medial wall), yielding 12 measurements per material group. Additionally, standard titanium meshes with thicknesses of 0.3 mm (Medartis AG, Basel, Switzerland) and 0.4 mm (Synthes Holding AG, Zuchwil, Switzerland) were assessed at the corresponding locations as reference materials.

**Fig. 2:**
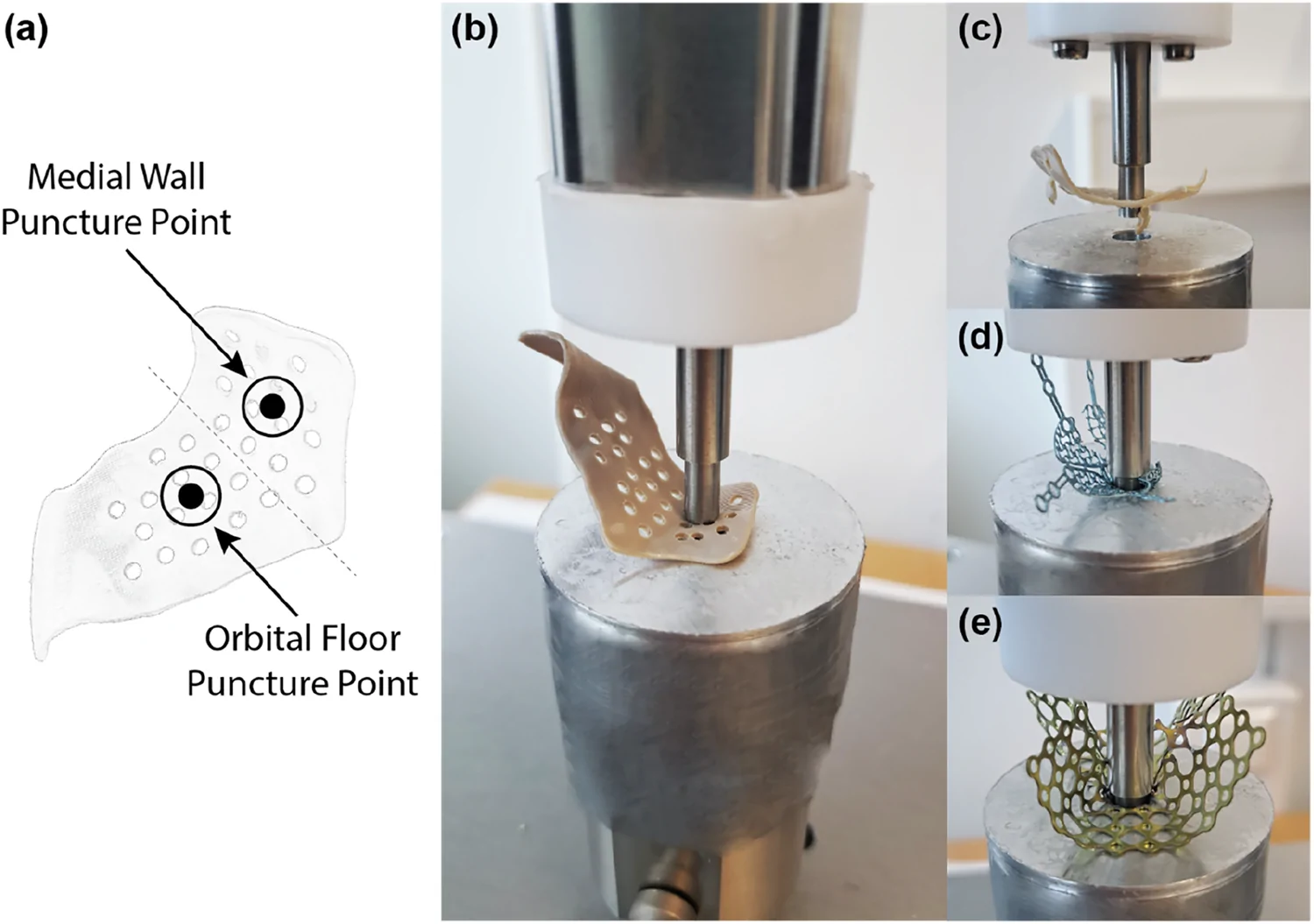
Biomechanical puncture strength testing setup and representative specimens. **a** Schematic diagram showing standardised puncture point locations on the orbital floor and medial wall of patient-specific orbital implants. **b** Representative fused filament fabrication (FFF) 3D-printed BaSO₄-PEEK implant positioned for puncture testing with a 5.5 mm diameter cylindrical punch. **c** Post-puncture test FFF BaSO₄-PEEK implant. **d** Titanium mesh specimen (0.3 mm, Medartis AG) during testing. **e** Post-puncture test titanium mesh (0.4 mm, Synthes Holding AG)

Puncture strength testing was conducted using a universal testing machine (Darto PM10, Darto GmbH, Wuppertal, Germany) equipped with a load cell and a cylindrical punch with a 5.5 mm diameter (cross-sectional area of 23.758 mm²). The punch diameter was selected based on established protocols for orbital floor biomechanical testing [26]. Each implant was positioned over a circular aperture, and the punch was placed at the centre of the orbital floor region (corresponding to the area of maximum orbital content pressure) for the first measurement point and at the flat surface of the medial wall for the second measurement point (Fig. 2a). Measurement locations were standardised across all samples. For FFF-fabricated implants, puncture testing locations were selected to align with the build orientation strategy: the orbital floor measurement point corresponded to loading perpendicular to the layer interfaces, whereas the medial wall measurement point corresponded to loading parallel to the layer interfaces.

Testing was performed under displacement control at 0.2 mm/s until implant failure, defined as complete puncture or a sudden drop in load-carrying capacity exceeding 20% (Fig. 2b–e). Load-displacement curves were recorded continuously. Peak puncture force (N) was determined as the maximum load sustained before failure, and puncture strength (N/mm²) was calculated by dividing peak force by this cross-sectional area. The effect of drainage hole diameter on mechanical performance was evaluated by comparing puncture strength between implants with 2 mm (*n* = 3 per material) and 3 mm (*n* = 3 per material) drainage holes for both unfilled PEEK and BaSO₄-PEEK formulations. This design enabled assessment of the impact of post-processing feature size on structural integrity, relevant to clinical customisation requirements. Magnified images of the fracture sites were acquired with a Leica DVM6 digital microscope fitted with a Leica PlanAPO FOV 43.75 low-magnification objective (Leica Microsystems, Wetzlar, Germany) and processed using Leica Application Suite X software (v3.0.11.20652, Leica Microsystems, Wetzlar, Germany).

### Cadaveric implantation and radiographic assessment

Nine fresh-frozen cadaveric heads (18 orbital sites) were obtained from Rise Labs (Benit 30A, 1043BB Amsterdam, Netherlands) with appropriate consent for research use. The study was conducted in accordance with institutional guidelines for cadaveric research, with ethical approval (Project-ID 2025-01141) obtained from the Ethics Commission of Northwestern and Central Switzerland (EKNZ). Each cadaveric head received two orbital implants of different materials, with material assignment and laterality randomised to ensure balanced distribution across specimens.

FFF 3D-printed orbital implants were positioned according to the virtual surgical plan without intraoperative contouring or modification and secured using two titanium microscrews (1.5 mm diameter × 6 mm length, SpeedTip, Medartis MODUS 2 Midface system, Medartis AG, Basel, Switzerland) placed through pre-designed screw holes. Titanium mesh implants underwent preoperative manual contouring using standard bending instruments and the corresponding 3D-printed anatomical biomodel as a template and were secured with two identical fixation screws. Final intraoperative adjustments included trimming the fixation bar and fine-tuning the contours to match the exposed bony margins. The orbital periosteum, extraocular muscles, and orbital fat pad were preserved during all procedures.

Postoperative CT scans were acquired using the same parameters as the preoperative scans. Radiopacity was quantitatively assessed using HU measurements with medical image processing software (Materialise Mimics Innovation Suite v. 26.0, Materialise NV, Leuven, Belgium). Three circular regions of interest (ROI, approximately 4 mm² each) were positioned on each implant at the anterior, middle, and posterior thirds. ROIs were sampled only from the implant material to avoid partial volume effects. Background reference measurements were obtained from orbital fat, extraocular muscles, and native cortical bone at the infraorbital rim. Image noise was calculated as the standard deviation of HU values in air outside the skull. Contrast-to-noise ratio (CNR) was calculated using the formula:

$${\rm{CNR}}=\frac{{\rm{mean\; implant\; HU}}-{\rm{mean\; background\; soft\; tissue\; HU}}}{{\rm{SD\; of\; noise\; measurement}}}$$

Reconstruction accuracy was evaluated by comparing the postoperative implant position with the preoperative virtual surgical plan. Since no preoperative virtual model of the intended bent geometry was available for the manually pre-bent titanium meshes, quantitative assessment of reconstruction accuracy was conducted exclusively for BaSO₄-PEEK patient-specific implants. Postoperative scans were co-registered with preoperative scans using registration algorithms. The postoperative implant was segmented from CT datasets using a threshold-based approach with manual refinement. Segmented implant surfaces were compared with the original virtual surgical plan using best-fit surface alignment and RMSE between planned and actual implant positions.

### Statistical analysis

All statistical analyses were performed using JASP (v. 0.95.4.0, University of Amsterdam, Amsterdam, Netherlands). Normality of data distribution was assessed using the Shapiro-Wilk test, and homogeneity of variance was assessed using Levene’s test. Continuous variables are reported as mean ± standard deviation. For dimensional accuracy, paired t-tests compared printed dimensions versus CAD specifications within each PEEK material group, and independent t-tests compared accuracy between unfilled PEEK and BaSO₄-PEEK groups. Reconstruction accuracy (RMSE between planned and actual implant positions) was compared between unfilled PEEK and radiopaque BaSO₄-PEEK using independent t-tests. HU measurements, CNR ratios, and biomechanical properties were compared among the three material groups (titanium mesh, unfilled PEEK, and BaSO₄-PEEK) using one-way ANOVA with Tukey’s post hoc test. Statistical significance was defined as *p* < 0.05 for all comparisons.

## Results

### Dimensional accuracy of FFF 3D-printed orbital implants

Dimensional accuracy analysis of six implants per material group revealed high geometric fidelity to original CAD designs for both FFF unfilled PEEK and FFF BaSO₄-PEEK (Table 2, Fig. 2). Overall RMSE was 0.31 ± 0.09 mm for FFF PEEK and 0.33 ± 0.08 mm for FFF BaSO₄-PEEK, with no significant difference between formulations (*p* = 0.77). Median deviations were 0.03 ± 0.04 mm for FFF PEEK and 0.03 ± 0.02 mm for FFF BaSO₄-PEEK.

**Table 2:** Dimensional accuracy of fused filament fabrication (FFF) 3D-printed unfilled polyetheretherketone (PEEK) and radiopaque PEEK orbital implants

	FFF PEEK	FFF BaSO₄-PEEK
**Deviation metrics**		
Minimum (mm)	−0.45 ± 0.03	−0.45 ± 0.04
Maximum (mm)	1.03 ± 0.16	1.44 ± 0.48
Median (mm)	0.03 ± 0.04	0.03 ± 0.02
RMSE (mm)	0.31 ± 0.09	0.33 ± 0.08
Surface within ±0.5 mm (%)	85.04 ± 10.95	84.72 ± 9.49
**Wall thickness measurements**		
Infraorbital Rim (mm)	0.80 ± 0.02	0.80 ± 0.01
Orbital floor (mm)	0.80 ± 0.02	0.81 ± 0.02
Medial wall (mm)	0.80 ± 0.01	0.79 ± 0.01
Overall mean thickness (mm)	0.80 ± 0.01	0.80 ± 0.01

Data presented as mean ± standard deviation. Target design thickness = 0.8 mm

*PEEK* polyetheretherketone, *BaSO*_*4*_ barium sulfate, *RMSE* root-mean-square error

All FFF-fabricated implants were successfully manufactured without print failures. Visual inspection revealed complete layer adhesion with no observable delamination, cracking, or void formation. Build time averaged 41 ± 2 min per implant following a 2.5-h chamber preheating period, with no difference in build duration between unfilled PEEK and BaSO₄-PEEK formulations.

**Fig. 3:**
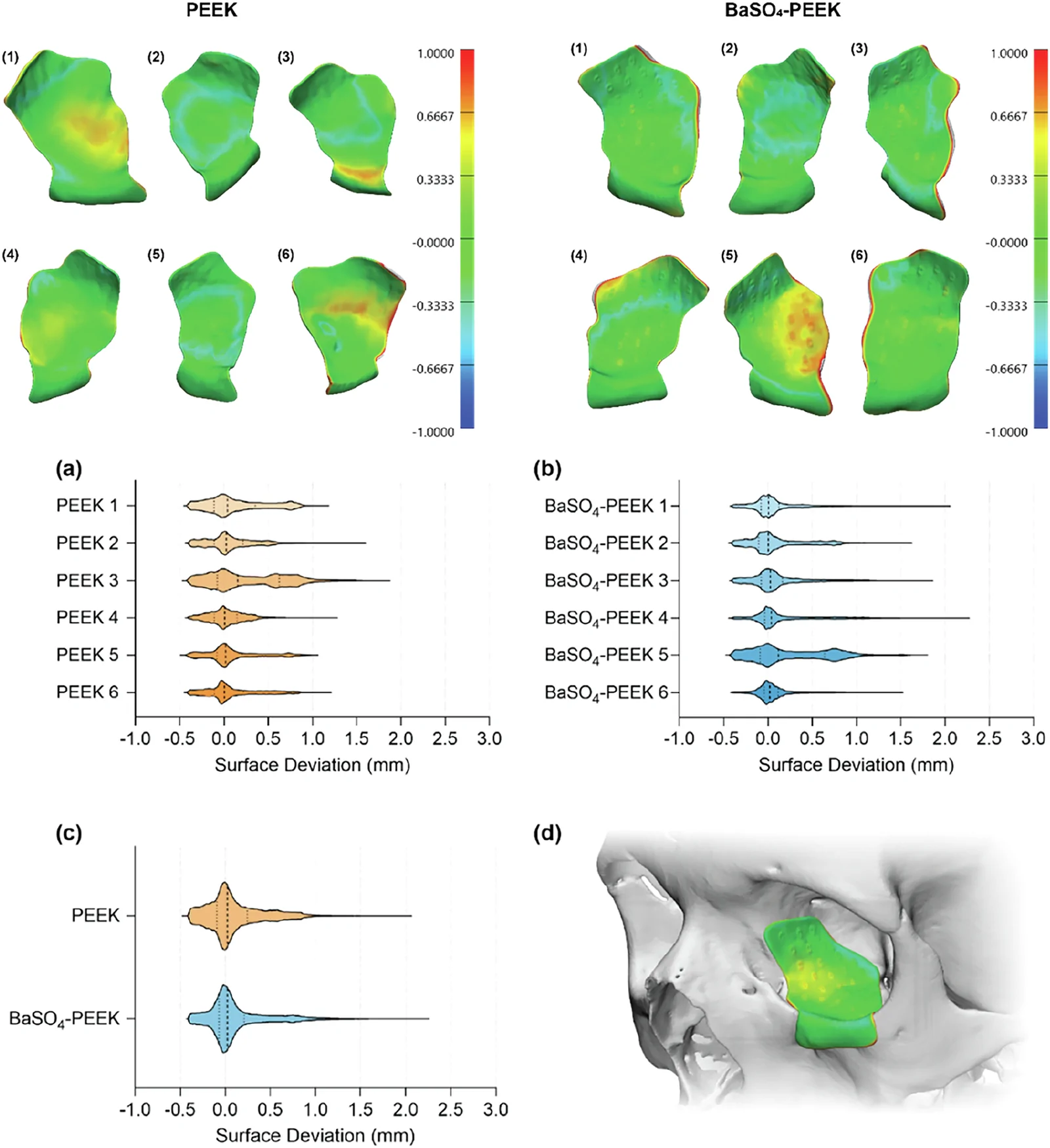
Dimensional accuracy analysis of fused filament fabrication (FFF) 3D-printed orbital implants. Colour-mapped deviation plots for six FFF unfilled PEEK implants (top left, specimens 1–6) and six FFF radiopaque BaSO₄-PEEK implants (top right, specimens 1–6) compared to original designs. The deviation scale ranges from −1.0 mm (blue) to +1.0 mm (red), with green indicating deviations within ±0.5 mm of the tolerance (representing 85% of the total surface area for both materials). Violin plots showing surface deviation distribution for (**a**) individual FFF unfilled PEEK specimen (*n* = 6, orange) and **b** individual FFF radiopaque BaSO₄-PEEK specimens (*n* = 6, blue) across the entire implant surface. **c** Comparison of pooled deviation distributions between FFF PEEK and FFF BaSO₄-PEEK formulations. **d** Representative colour-mapped implant positioned on an anatomical biomodel illustrating deviation analysis in a clinical context

Colour-mapped deviation analysis across all specimens (Fig. 3) showed that 85.04 ± 10.95% of FFF PEEK implant surface area and 84.72 ± 9.49% of FFF BaSO₄-PEEK surface area fell within ±0.5 mm tolerance. Violin plot analysis (Fig. 3a-c) demonstrated consistent deviation distributions across individual specimens within each material group. Maximum positive deviations of 1.03 ± 0.16 mm (FFF PEEK) and 1.44 ± 0.48 mm (FFF BaSO₄-PEEK) were localised to support-structure interface regions (<5% of total surface area), predominantly at the medial wall margin and infraorbital rim, where support density was highest (Fig. 3d). Maximum negative deviations were −0.45 ± 0.03 mm for FFF PEEK and −0.45 ± 0.04 mm for FFF BaSO₄-PEEK.

Wall thickness measurements at three standardised anatomical locations are presented in Table 2. FFF PEEK orbital implants exhibited mean thicknesses of 0.80 ± 0.02 mm at the infraorbital rim, 0.80 ± 0.02 mm at the orbital floor, and 0.80 ± 0.01 mm at the medial wall. FFF BaSO₄-PEEK orbital implants demonstrated mean thicknesses of 0.80 ± 0.01 mm at the infraorbital rim, 0.81 ± 0.02 mm at the orbital floor, and 0.79 ± 0.01 mm at the medial wall. The overall measured thickness was 0.80 ± 0.01 mm for both formulations.

### Biomechanical performance

**Fig. 4:**
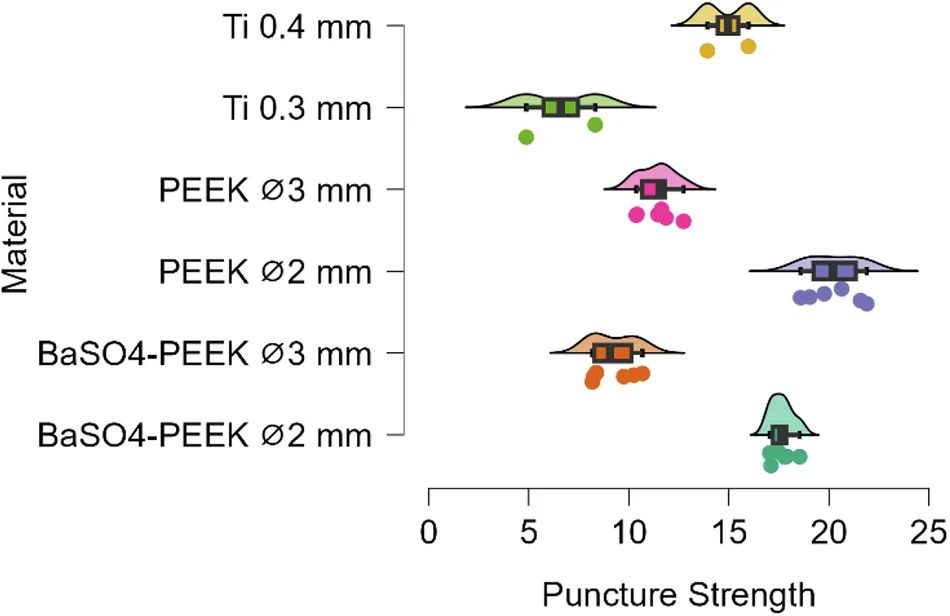
Puncture strength comparison of fused filament fabrication (FFF) 3D-printed orbital implant materials. Bar chart showing mean puncture strength (N/mm²) for titanium mesh, FFF unfilled PEEK, and FFF BaSO₄-PEEK at orbital floor and medial wall measurement points. Error bars represent the standard deviation

**Fig. 5:**
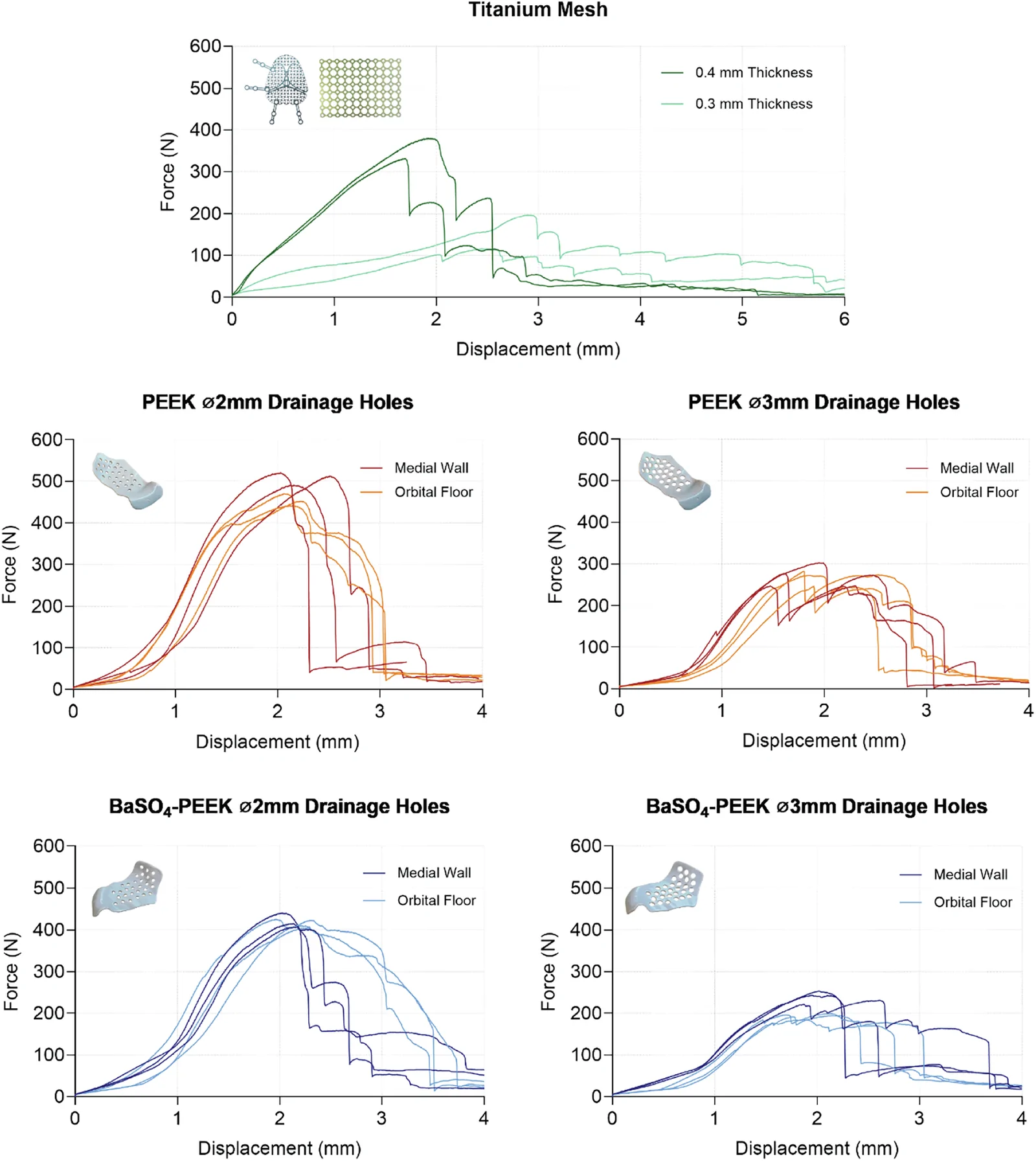
Load-displacement curves during puncture strength testing. Top row: Representative curves for titanium meshes showing 0.4 mm thickness (dark line) and 0.3 mm thickness (light line). Insets show mesh structure and specimen configuration. Middle row: Fused filament fabrication (FFF) 3D-printed unfilled PEEK implants with 2 mm drainage holes (left) and 3 mm drainage holes (right), showing medial wall (red lines) and orbital floor (orange lines) measurements. Bottom row: FFF BaSO₄-PEEK implants with 2 mm drainage holes (left) and 3 mm drainage holes (right), showing medial wall (dark blue lines) and orbital floor (light blue lines) measurements. Each panel shows multiple test replicates. Peak forces occurred at displacements of 1.8–2.7 mm across all materials. Insets illustrate implant geometry and drainage hole configuration

Puncture strength testing revealed significant differences in load-bearing capacity among materials and anatomical sites (Table 3, Figs. 4,5). One-way ANOVA demonstrated significant differences among material groups (*p* < 0.001).

**Table 3:** Puncture strength analysis of fused filament fabrication (FFF) 3D-printed orbital implant materials at two anatomical measurement sites

	Puncture area	Peak force (N)	Displacement at failure (mm)	Puncture strength (N/mm^2^)
**Ti (0.3** **mm)**	Orbital floor	156.81 ± 57.86	2.67 ± 0.33	6.60 ± 2.44
**Ti (0.4** **mm)**	Orbital floor	355.29 ± 34.37	1.82 ± 0.18	14.95 ± 1.45
**FFF PEEK** **(⌀2** **mm)**	Medial wall	507.66 ± 15.53	2.22 ± 0.25	21.37 ± 0.65
	Orbital floor	454.55 ± 14.16	2.15 ± 0.08	19.13 ± 0.60
**FFF PEEK** **(⌀3** **mm)**	Medial wall	275.37 ± 27.71	1.95 ± 0.35	11.59 ± 1.17
	Orbital floor	266.82 ± 18.27	1.85 ± 0.05	11.23 ± 0.77
**FFF** **BaSO**_**4**_**-PEEK** **(⌀2** **mm)**	Medial wall	420.57 ± 17.72	2.10 ± 0.08	17.70 ± 0.75
	Orbital floor	417.56 ± 10.95	2.17 ± 0.19	17.58 ± 0.46
**FFF** **BaSO**_**4**_**-PEEK** **(⌀3** **mm)**	Medial wall	243.03 ± 11.00	2.19 ± 0.36	10.23 ± 0.46
	Orbital floor	196.48 ± 2.41	2.00 ± 0.25	8.27 ± 0.10

Data presented as mean ± standard deviation. Titanium meshes were evaluated at the orbital floor location only. Punch diameter = 5.5 mm (23.758 mm² cross-sectional area)

*Ti* titanium mesh, *PEEK* polyetheretherketone, *BaSO*_*4*_ barium sulfate, *FFF* fused filament fabrication, *⌀* drainage hole diameter

#### Material comparison

For the orbital floor measurement point with 2 mm drainage holes, the mean puncture strength was 19.13 ± 0.60 N/mm² for FFF PEEK and 17.58 ± 0.46 N/mm² for FFF BaSO₄-PEEK (Fig. 4). FFF BaSO₄-PEEK exhibited significantly lower puncture strength than FFF PEEK (*p* = 0.008). FFF BaSO₄-PEEK with 2 mm drainage holes (17.58 ± 0.46 N/mm²) showed no significant difference compared to 0.4 mm titanium mesh (14.95 ± 1.45 N/mm², *p* = 0.083).

#### Drainage hole effect

Drainage-hole diameter significantly affected puncture strength in both PEEK formulations (Fig. 4). Mean puncture strength with 2 mm holes was 19.13 N/mm² for FFF PEEK and 17.58 N/mm² for FFF BaSO₄-PEEK, compared to 11.23 N/mm² and 8.27 N/mm² with 3 mm holes, respectively (*p* < 0.001 for both). FFF BaSO₄-PEEK with 3 mm drainage holes (8.27 ± 0.10 N/mm²) showed no significant difference compared to 0.3 mm titanium mesh (6.60 ± 2.44 N/mm², *p* = 0.090).

#### Anatomical site comparison

Medial wall puncture strength exceeded orbital floor values across all materials (Table 3). For FFF PEEK with 2 mm holes, medial wall strength was 21.37 ± 0.65 N/mm² versus 19.13 ± 0.60 N/mm² for the orbital floor. For FFF BaSO₄-PEEK with 2 mm holes, values were 17.70 ± 0.75 N/mm² versus 17.58 ± 0.46 N/mm², respectively.

Mean displacement at peak force ranged from 1.82 to 2.67 mm across all material groups (Fig. 4), with no significant differences observed among materials or anatomical sites (*p* > 0.05).

#### Failure modes differed across materials

**Fig. 6:**
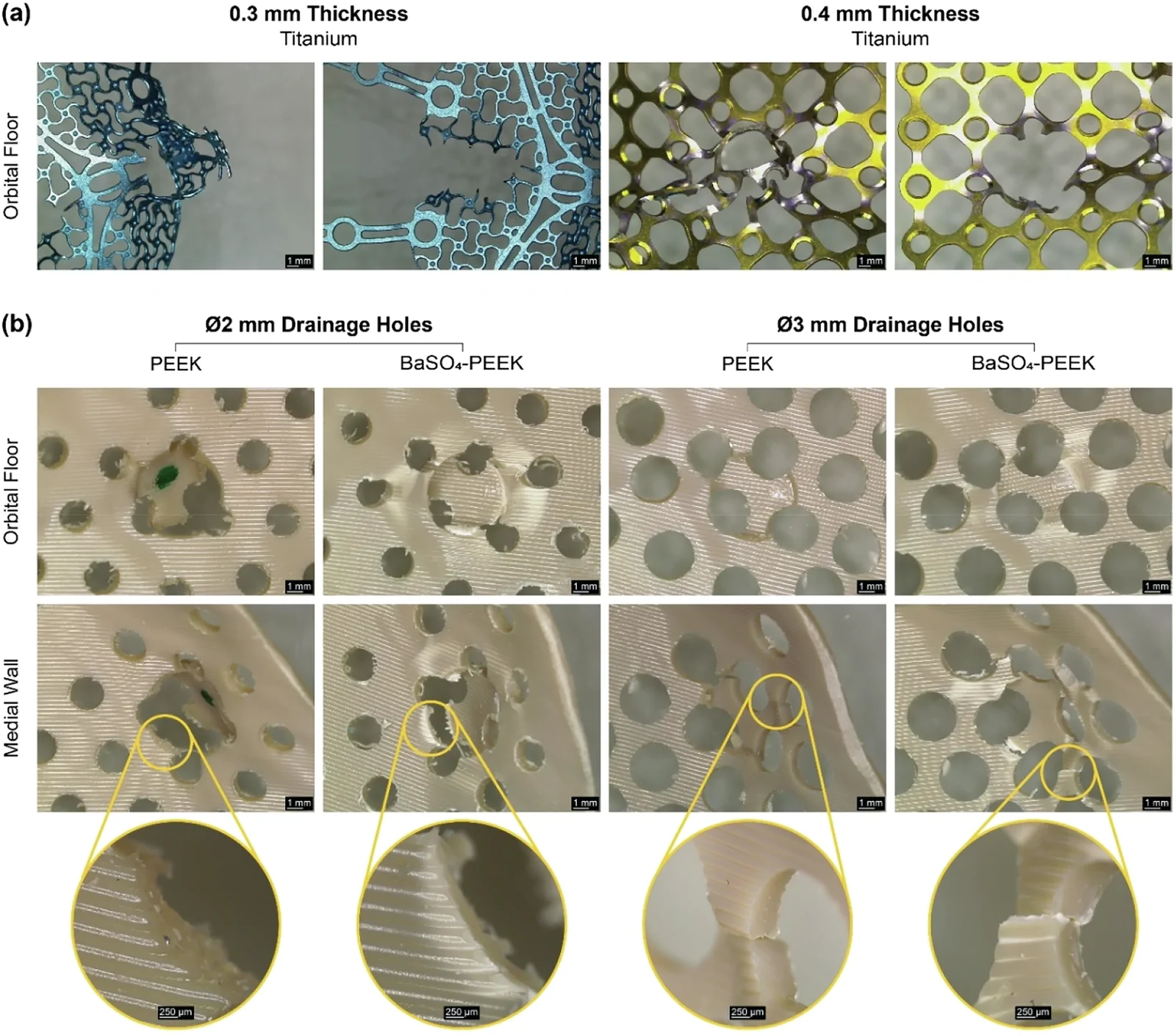
Digital microscope images of fracture sites in titanium orbital meshes and Fused Filament Fabrication (FFF) PEEK PSIs following puncture strength testing. **a** Titanium meshes of 0.3 mm (left) and 0.4 mm (right) thickness, showing strut tearing and ductile deformation in the orbital floor region. **b** Unfilled PEEK and BaSO₄-PEEK PSIs with ⌀2 mm (left) and ⌀3 mm (right) drainage holes, showing fractures in the orbital floor and medial wall regions. Magnified detail views (×225) of the reveal clean layer-aligned fracture edges in unfilled PEEK and pronounced stress-whitening in BaSO₄-PEEK, indicative of filler-matrix debonding. All overview images were acquired at ×52 magnification; scale bars represent 1 mm (overview) and 250 μm (detail views)

Titanium meshes exhibited tearing and bending of the mesh struts at the puncture site for both 0.3 mm and 0.4 mm thicknesses, without complete material separation (Fig. 6a). Both FFF unfilled PEEK and FFF BaSO₄-PEEK implants demonstrated limited plastic deformation prior to failure, with linear fracture propagation aligned with the build orientation and no interlayer delamination (Fig. 6b). Fracture consistently initiated at the drainage holes, with more extensive material loss observed in ⌀3 mm hole configurations than in ⌀2 mm configurations. The magnified detail views (×225) revealed distinct fracture surface morphologies: unfilled PEEK exhibited clean fracture edges with visible layer lines, whereas BaSO₄-PEEK showed pronounced stress-whitening at the fracture site.

### Radiopacity and imaging performance

**Fig. 7:**
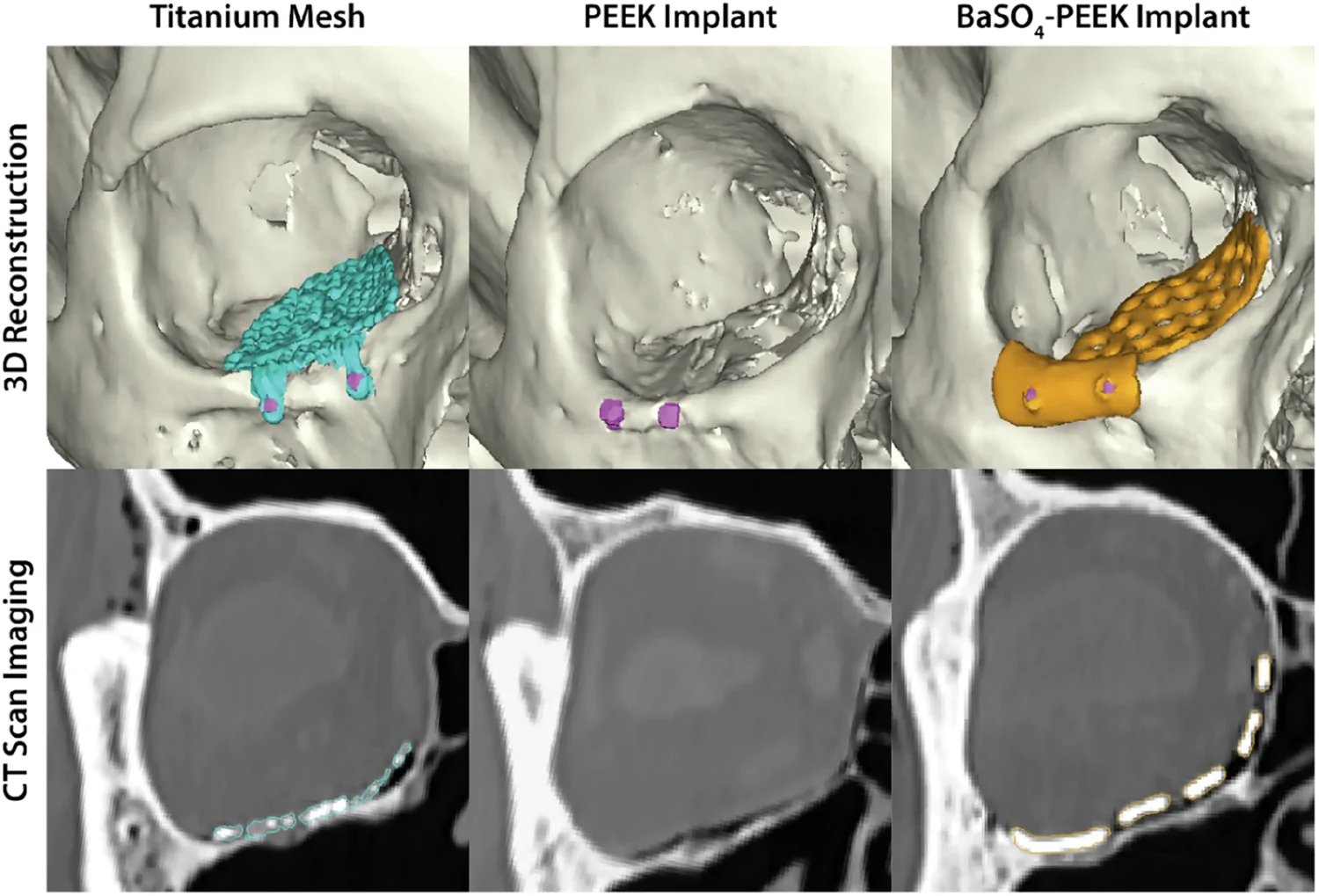
Radiographic visibility of fused filament fabrication (FFF) 3D-printed orbital implant materials on postoperative computed tomography (CT) imaging. Top row: Three-dimensional (3D) reconstructions showing titanium mesh (left), FFF polyetheretherketone (PEEK) (middle), and FFF BaSO₄-PEEK (right) orbital implants in situ with fixation screws. Bottom row: Corresponding coronal CT slices demonstrating titanium mesh with metal artefacts (left), radiolucent unfilled PEEK implant (middle, not visible), and radiopaque BaSO₄-PEEK implant (right) with clear margin delineation and minimal artefacts

**Fig. 8:**
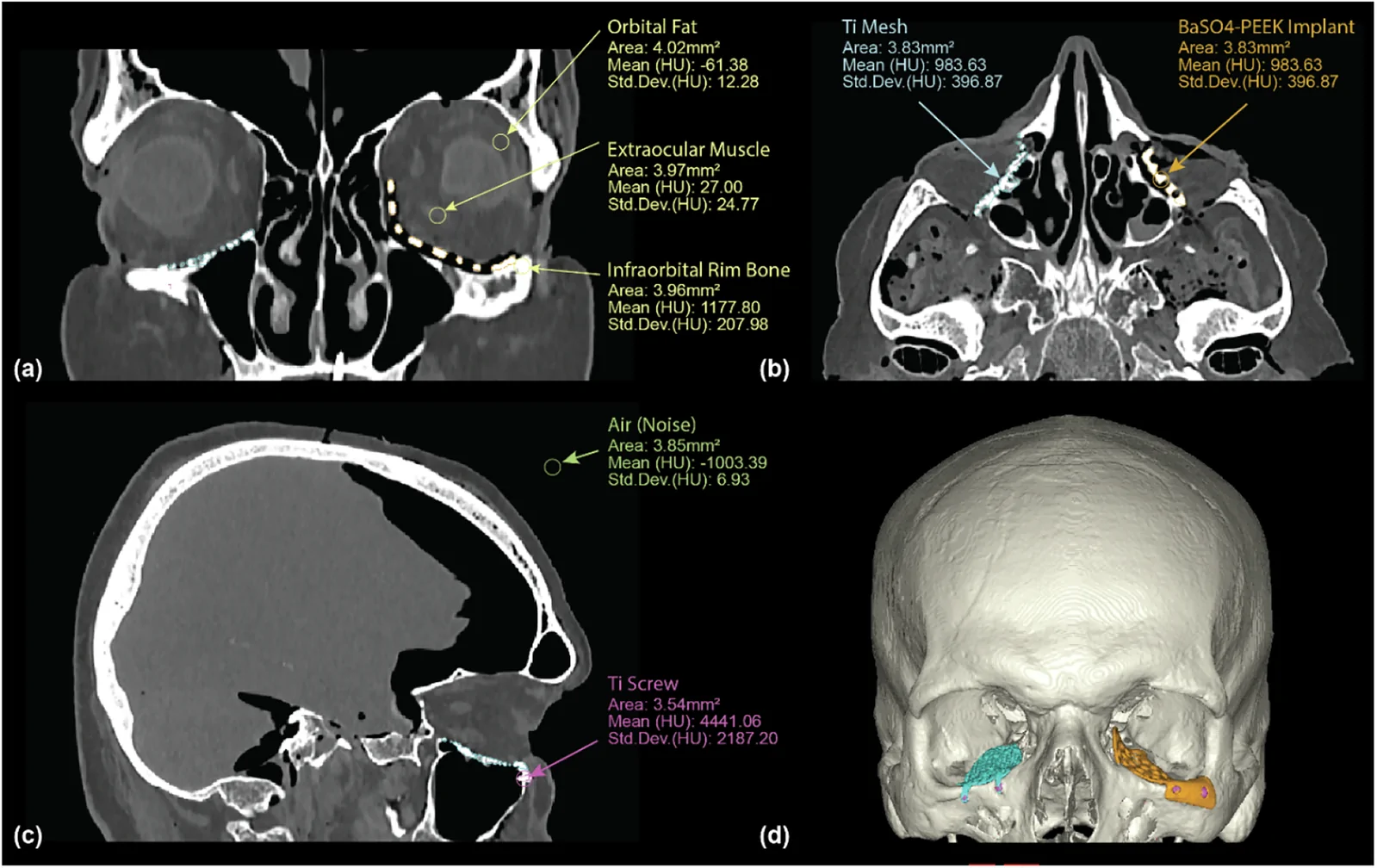
Hounsfield Unit (HU) measurements and anatomical reference structures. **a** Coronal CT slice showing regions of interest (ROI) for background measurements: orbital fat, extraocular muscle, and infraorbital rim bone. **b** Axial CT slice demonstrating ROI placement on titanium mesh and fused filament fabrication (FFF) BaSO₄-PEEK implants. **c** Sagittal CT slice showing a titanium fixation screw and an air (noise) measurement ROI. **d** 3D reconstruction illustrating bilateral implant placement with titanium mesh (left, cyan) and FFF BaSO₄-PEEK (right, orange) for direct visual comparison

HU measurements from cadaveric CT scans demonstrated distinct radiographic characteristics for each material (Table 4, Figs. 7,8). Mean HU values were 2467 ± 307 HU for FFF BaSO₄-PEEK and 1767 ± 48 HU for titanium mesh across three measurement regions (anterior, middle, posterior). FFF unfilled PEEK orbital implants were entirely radiolucent and could not be visualised or quantified on CT scans.

**Table 4:** Hounsfield unit (HU) measurements and contrast-to-noise ratios (CNR) for fused filament fabrication (FFF) 3D-printed orbital implant materials

	Anterior HU	Middle HU	Posterior HU	Mean HU	CNR
Ti mesh	1760.20	1818.98	1722.89	1767.36 ± 48.44	673.83
FFF PEEK	x	x	x	x	x
FFF BaSO_4_-PEEK	2271.83	2307.26	2820.83	2466.64 ± 307.25	930.97
Screws				4425.49 ± 988.72	
Orbital fat				−77.72 ± 15.45	
Extraocular muscle				29.44 ± 9.15	
Infraorbital rim bone				1126.13 ± 161.40	
Air (Noise)				−1001.79 ± 2.73	

*Ti* titanium mesh, *PEEK* polyetheretherketone, *BaSO*_*4*_ barium sulfate, *FFF* fused filament fabrication, *HU* hounsfield units, *CNR* contrast-to-noise ratio, × not visible/not quantifiable

CNR calculations comparing implant materials to orbital soft tissue yielded values of 931 for FFF BaSO₄-PEEK and 674 for titanium mesh (Fig. 8). Background reference measurements yielded −78 ± 15 HU for orbital fat, 29 ± 9 HU for extraocular muscles, and 1126 ± 161 HU for cortical bone at the infraorbital rim.

Postoperative CT imaging showed that FFF BaSO₄-PEEK implants were clearly visible across all imaging planes, whereas titanium mesh produced metal artefacts that obscured adjacent soft-tissue structures (Fig. 7). FFF PEEK implants remained invisible on all imaging planes.

### Reconstruction accuracy and surgical placement assessment

**Fig. 9:**
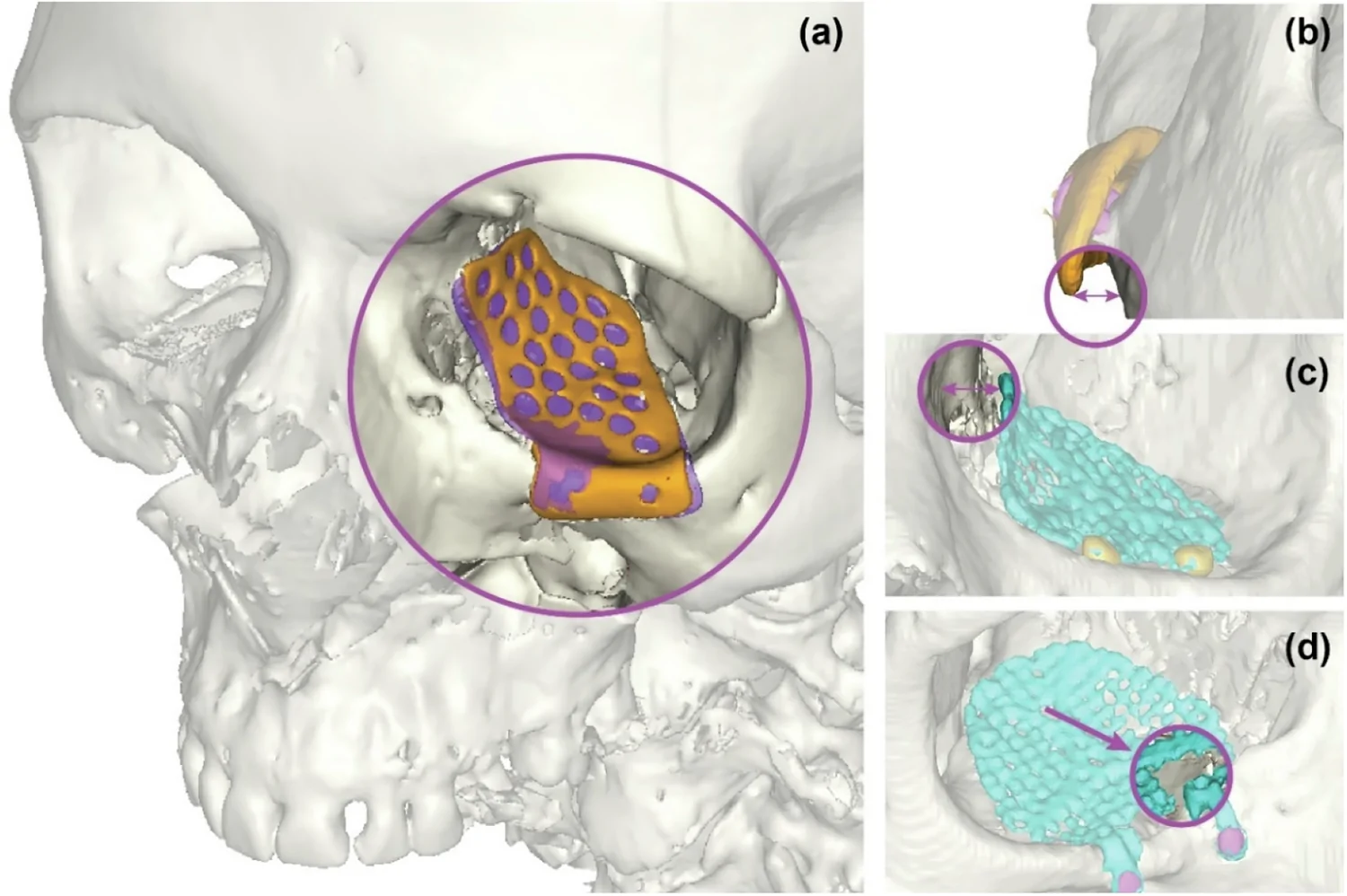
Reconstruction accuracy assessment of fused filament fabrication (FFF) 3D-printed BaSO₄-PEEK orbital implants. **a** Overlay of planned (orange wireframe) and actual (purple solid) FFF BaSO₄-PEEK implant position with magnified inset. Representative postoperative computed tomography (CT) findings: **b** FFF BaSO₄-PEEK implant with gap at infraorbital rim (purple circle), **c** Titanium mesh with gap at medial orbital wall (purple circle), and **d** titanium mesh with metal artefacts (arrow, purple circle) limiting visualisation of implant position

Quantitative 3D reconstruction accuracy assessment comparing postoperative implant position with preoperative virtual surgical plans was performed exclusively for FFF BaSO₄-PEEK orbital implants (Fig. 9a). This analysis was not feasible for FFF unfilled PEEK orbital implants because radiolucency precluded postoperative CT visualisation. Titanium mesh implants were pre-fabricated stock implants without corresponding digital design files, precluding quantitative dimensional comparison. Overlay analysis of planned versus actual FFF BaSO₄-PEEK implant position yielded a mean RMSE of 0.61 ± 0.15 mm (range: 0.42–0.82 mm).

Qualitative postoperative CT evaluation identified anatomical gaps in 1 of 6 FFF BaSO₄-PEEK cases (at the infraorbital rim, Fig. 9b) and 1 of 6 titanium mesh cases (at the medial orbital wall, Fig. 9c). Metal artefacts from titanium mesh implants obscured implant-bone interfaces in titanium specimens (Fig. 9d), while FFF BaSO₄-PEEK implants demonstrated clear margin delineation without imaging artefacts.

## Discussion

The dimensional accuracy achieved for BaSO₄-PEEK composites in thin-walled orbital implants demonstrates that adding 20 wt% inorganic filler does not significantly affect geometric precision. The lack of notable differences between unfilled PEEK and BaSO₄-PEEK in dimensional performance challenges common assumptions about the processability of filled polymers, where increased melt viscosity is often associated with reduced dimensional control. This maintained accuracy results from several material-process interactions. First, BaSO₄ particles at 20 wt% increase melt viscosity by about 40–60% compared to unfilled PEEK, but this remains manageable when nozzle temperatures are raised to ensure proper flow. Second, the high thermal conductivity of BaSO₄ helps dissipate local heat, reducing temperature gradients during layer deposition. Third, the rigid particle network may limit polymer chain mobility during cooling, reducing warping issues that often affect thin-walled PEEK parts.

The uniformity of wall thickness observed across all anatomical regions for both formulations indicates even material deposition and consistent interlayer adhesion despite complex geometry. This consistency is essential for thin-walled structures, where small dimensional changes can weaken them or affect anatomical fit. The localised deviations at support-structure interfaces are due to post-processing artefacts rather than inherent material flaws, as evidenced by their concentration in peripheral areas requiring manual finishing. Developing optimised support removal methods, such as 3D-printed post-processing guides or dissolvable support materials compatible with high-temperature processing, could further reduce these artefacts. Similar dimensional accuracy across formulations suggests that a 20 wt% filler concentration strikes a good balance between radiopacity and processability, in contrast to reports of dimensional issues at higher loadings (>30 wt%) [21]. This shows that BaSO₄-PEEK at 20 wt% remains processable in complex geometries, unlike unfilled polymers, which often exhibit dimensional warping. The quick build times, combined with a high success rate across both formulations, demonstrate process reliability, supporting FFF as a feasible method for producing patient-specific polymer composite parts with the potential for geometric customisation [27].

The mechanical performance of BaSO₄-PEEK composites reflects the opposing effects of inorganic filler addition. The slight decrease in strength relative to unfilled PEEK indicates that rigid BaSO₄ particles create stress concentrations that can cause local yielding. However, this effect is much smaller than what simple composite mechanics would predict for systems with non-bonded filler particles. This indicates that adequate particle-matrix bonding occurs during melt processing, where higher temperatures promote close contact between molten PEEK and BaSO₄ particles, forming hydrogen bonds or van der Waals interactions at the interface. The retention of performance at 20 wt% loading suggests this is an optimal balance point. These lower amounts would result in reduced radiopacity in clinical imaging, while higher amounts would significantly increase melt viscosity and the risk of particle clustering, creating larger stress concentration zones and further weakening mechanical integrity. When compared with published data indicating that human orbital floor bone has much lower puncture strength and that orbital contents exert minimal static stress [26, 28], the tested materials offer considerable safety margins that exceed physiological needs.

Feature-based mechanical analysis showed that post-processing feature size significantly affects load-bearing capacity, with larger drainage holes decreasing puncture strength. This indicates that stress concentration scales with feature size in thin-walled structures, particularly as the ratio of hole diameter to wall thickness approaches critical levels at which stress singularity formation occurs. From a design standpoint, these results provide quantitative guidelines suggesting that post-processing features should be minimised in size and positioned away from high-stress areas to maintain structural performance. The linear relationship between hole diameter and strength loss enables predictive design adjustments, allowing feature size to be tailored to meet specific mechanical needs.

The observed failure modes, with controlled linear fracture propagation aligned with the build orientation and without delamination or catastrophic fragmentation, indicate adequate interlayer bonding despite altered melt rheology. The stress-whitening observed at BaSO₄-PEEK fracture sites, absent in unfilled PEEK, is indicative of filler-matrix interface debonding and localised plastic deformation, suggesting a more ductile failure mode. This is consistent with the slightly lower peak force exhibited by BaSO₄-PEEK, indicating that the filler-matrix interface enables energy absorption through localised deformation rather than purely brittle failure. Fracture initiation at the drainage holes, more pronounced with larger ⌀3 mm features, reflects the greater stress concentration associated with increased feature size in thin-walled structures. The consistent linear, build-aligned fracture in both formulations differs from the brittle stellate fragmentation typical of injection-moulded polymers, offering a safety advantage for structural applications where catastrophic fragmentation could produce sharp fragments. While the observed performance suggests sufficient interlayer bonding under the tested conditions, testing across multiple orientations would provide a more comprehensive understanding of directional properties in FFF PEEK composites [29–32].

The radiographic performance of BaSO₄-PEEK composites demonstrates effective radiopacity while avoiding the metal artefacts that hinder imaging of titanium mesh. The improved CNR ratio compared to titanium results from fundamental differences in X-ray interaction mechanisms: the atomic number and density of BaSO₄ provide strong X-ray attenuation via photoelectric absorption, while the polymer matrix holds BaSO₄ particles in fixed positions, preventing surface electron-photon interactions that cause streak artefacts in metallic implants. Measurements across anterior, middle, and posterior regions confirm even BaSO₄ dispersion in the commercial filament and no filler redistribution during processing. This is crucial for both radiographic performance and mechanical strength, as particle agglomeration can create localised regions of varying radiopacity, potentially weakening the structure. Such spatial uniformity is essential for reliable radiographic imaging, providing consistent visibility across complex shapes and maintaining mechanical integrity by preventing filler-rich areas that increase brittleness or filler-depleted zones that diminish radiopacity. Beyond artefact elimination, BaSO₄-PEEK offers the additional clinical advantage of avoiding metal-related hypersensitivity reactions. Although titanium hypersensitivity is relatively uncommon, it has been reported to manifest as local inflammation, eczema, or implant failure, and represents a clinical consideration particularly relevant for patients with a history of metal sensitivities [33].

While PEEK demonstrates excellent biocompatibility and chemical inertness, it inherently lacks osteoconductive properties, limiting direct bone bonding compared to titanium. PEEK typically elicits fibrous tissue encapsulation rather than direct bone apposition, which may necessitate surface modifications or porous architectures to enhance osseointegration in load-bearing applications [34, 35]. However, in orbital wall reconstruction, implant stability relies primarily on screw fixation rather than osseointegration, making this limitation less clinically significant than in weight-bearing orthopaedic applications.

The selection of 20 wt% BaSO₄ concentration represents an optimised balance across multiple performance areas through multi-objective material design. Radiographically, this loading provides attenuation values well above the threshold needed for clinical visualisation and margin delineation. Mechanically, it maintains most of the strength of unfilled PEEK. From a processability standpoint, the increase in melt viscosity remains manageable with elevated temperature, without requiring specialised high-shear extrusion equipment. Higher loadings would offer diminishing radiographic benefits as attenuation plateaus and would complicate processing through excessive viscosity and particle clustering. This illustrates a key principle for functional polymer composites: the optimal formulation provides sufficient performance across all requirements rather than maximising any single property.

The quantitative reconstruction accuracy shows that manufacturing dimensional precision directly translates to functional performance in precision-fit applications. The submillimetre positioning accuracy confirms that the geometric fidelity achieved during fabrication enables precise implementation of digital designs. This represents a shift away from traditional methods that use manually bent titanium mesh, which is operator-dependent, time-consuming, and susceptible to positional errors [36, 37]. The implant design parameters in this study, including the 0.8 mm wall thickness, were informed by a previously published multi-criteria assessment framework that evaluated FFF-fabricated PEEK orbital implants through finite element analysis of stress and deformation alongside morphological fit [38]. Implant configurations at or above this thickness have previously been shown to achieve optimal performance scores by balancing stress distribution and morphological fit. The elastic modulus of PEEK, which more closely approximates that of cortical bone than titanium, reduces stress shielding at fixation points and may lower the risk of bone resorption adjacent to screw fixation sites. Furthermore, FFF enables customisation of implant geometry and porosity to match the complex thin-walled anatomy of the orbital floor, supporting simultaneous optimisation of mechanical performance, radiographic visibility, and anatomical conformity for individual patients. However, gaps observed in some cases highlight that placement technique and operator skill remain vital to achieving optimal fit, regardless of manufacturing precision [39].

The comparison between patient-specific FFF BaSO₄-PEEK implants and manually contoured titanium mesh reveals fundamental differences in manufacturing approaches. Manual contouring introduces operator-dependent geometric variability, compromising dimensional consistency, while stock components limit geometric customisation to basic shape adaptation and preclude patient-specific optimisation. Unlike subtractive manufacturing, which requires custom tooling and generates material waste, or injection moulding, which requires high-volume production for cost efficiency, FFF enables on-demand fabrication of customised geometries with minimal setup while maintaining filler uniformity through controlled extrusion [40]. This manufacturing flexibility is particularly valuable for applications in which geometric variation across cases precludes the use of standardised component designs. The radiopaque polymer formulation further demonstrates that FFF can incorporate functional materials that are difficult to process using traditional methods. Injection moulding of filled PEEK requires precise control of filler distribution during mould filling, whereas machining removes material regardless of functional requirements [41]. FFF maintains filler uniformity through controlled extrusion, enabling simultaneous optimisation of geometry and material properties [42]. This combination of processes, structures, and properties highlights a key advantage of FFF in producing functional components. From a materials-processing perspective, the demonstrated performance confirms that FFF is a viable production method for functional composite structures in medical device applications, within the scope of the system and formulation studied.

The thermal stability of PEEK composites allows compatibility with standard steam sterilisation protocols, enabling FFF 3D-printed personalised implants to be produced, quality-checked, and sterilised without losing their shape or structure [43, 44]. This sterilisation compatibility is crucial for integrating into clinical workflows and aligns with established methods for processing polymer medical devices. The thorough assessment across size, strength, imaging, and function shows several universal principles for filler-reinforced polymers in precise engineering. First, multi-objective optimisation, by balancing competing needs, contrasts with traditional designs that focus on single metrics, underscoring the importance of comprehensive testing across all relevant performance areas. For functional composites, the best formulation usually does not maximise a single property but instead provides sufficient performance across all required aspects. Second, changes in flow behaviour caused by filler addition can be managed through systematic processing adjustments rather than indicating fundamental material limits. Adjusting processing parameters with more thermal energy enables the use of functional fillers that might otherwise be incompatible with standard processing conditions. Third, manufacturing personalised medical devices with thin walls requires careful control of material-processing interactions due to increased thermal gradients, reduced stiffness during manufacturing, and greater susceptibility to residual stresses. Proper thermal management, such as higher chamber temperatures and controlled cooling, can address the geometric scaling issues that usually limit the smallest manufacturable dimensions. Fourth, for precision applications, achieving manufacturing accuracy alone does not ensure functional success; the entire process, from material selection through processing and application, must maintain geometric accuracy.

Future optimisation pathways include systematically investigating different filler concentrations with detailed rheological and mechanical analyses to establish process-property relationships, evaluating alternative radiopaque fillers, optimising particle size, and developing spatially graded composites for region-specific property optimisation. Advanced processing strategies could further enhance performance through multi-material deposition, enabling discrete transitions in filler concentration within a single component, in situ annealing during layer deposition to improve crystallinity and reduce residual stress, and adaptive temperature control based on local geometry. Surface modification strategies such as plasma activation, hydroxyapatite coating, or natural antibacterial coatings may improve biological integration and reduce the risk of infection while remaining compatible with FFF processes [45, 46]. Additionally, automated quality-control methods based on in-process monitoring could improve manufacturing consistency in patient-specific production [47].

This study focused on functional validation for clinical translation rather than comprehensive materials characterisation. Detailed microstructural analysis and additional mechanical testing modalities would provide a deeper understanding of structure-property relationships, but were beyond the scope of this device validation study. The selected approach prioritises clinically relevant performance metrics such as dimensional accuracy, puncture resistance under physiological loading, radiographic visibility, and surgical placement precision that directly inform translational pathways for patient-specific medical devices.

Several limitations merit consideration. Evaluating a single filler concentration limits understanding of concentration-response relationships, which could identify alternative optimal points for different performance criteria. A parametric study varying filler content would establish quantitative relationships among filler concentration, dimensional accuracy, mechanical properties, and radiographic performance, thereby supporting application-specific formulation optimisation. Mechanical testing employed quasi-static loading to simulate single-event failure scenarios; dynamic fatigue behaviour under cyclic physiological loading remains unexamined. Additionally, the mechanical anisotropy inherent to FFF processing was not quantified, as puncture testing was performed at standardised anatomical locations that reflect clinically relevant loading conditions rather than across the principal build directions. Future studies should evaluate orientation-dependent mechanical properties to fully characterise the anisotropic behaviour of FFF-fabricated BaSO₄-PEEK. Furthermore, post-processing steps, including drilling drainage holes and surface finishing, introduce potential stress concentrators and remove as-printed surface characteristics. The anatomical gap observed in one BaSO₄-PEEK case cannot be attributed to a single cause, as the current study design does not allow isolation of manufacturing deviation, post-processing variability, or surgical placement error as contributing factors. Future studies incorporating pre- and post-processing dimensional assessment would enable differentiation of these individual contributions. Lastly, the cadaveric model allows controlled evaluation but cannot replicate in vivo biological responses, such as tissue integration, inflammatory reactions, or long-term material degradation. While the base material is known to be biocompatible, the specific filled formulation requires biological validation through animal studies or clinical trials. Finally, the applicability of the findings is specific to the current FFF system (printer and filament) used in this study, and transferability to other FFF platforms or alternative radiopaque PEEK formulations with different filler types, concentrations, or particle morphologies requires independent validation.

## Conclusion

This study demonstrates that FFF BaSO₄-PEEK composites with a 20 wt% filler content are effective, high-performance materials for thin-walled medical implants. It shows that adding functional filler can enhance radiographic properties without significantly affecting dimensional accuracy, mechanical performance, or processability. Dimensional analysis indicates that geometric fidelity is maintained in submillimetre wall structures, suggesting that inorganic fillers preserve precision like unfilled polymers despite a significant increase in melt viscosity. Mechanical testing confirms that the material exhibits adequate structural performance, maintaining high strength and displaying controlled failure modes, indicating good interlayer bonding. Radiographic evaluation reveals a notable improvement in CNR compared with metallic options, and the polymer-based composition eliminates metal artefacts. Functional validation through cadaveric surgical implantation confirms that manufacturing precision results in accurate application, with submillimetre positioning accuracy. These findings show that optimal filler levels balance performance requirements, while processing adjustments mitigate rheological changes caused by fillers. Proper thermal management is essential for thin-walled designs, and manufacturing precision ensures performance by maintaining dimensional accuracy throughout processing. These insights establish a framework for fabricating FFF functional composites, systematically balancing multiple criteria to meet specific application demands in medical, aerospace, and biomedical fields where accuracy, reliability, and properties must be optimised.

## Data Availability

Data will be made available on request.

## Abbreviations

3D: Three Dimensional
AM: Additive Manufacturing
BaSO_4_: Barium Sulfate
CAD: Computer-Aided Design
CNC: Computer Numerical Control
CNR: Contrast-to-Noise Ratio
CT: Computed Tomography
DICOM: Digital Imaging and Communications in Medicine
FFF: Fused Filament Fabrication
HU: Hounsfield Unit
ICP: Iterative Closest Point
MRI: Magnetic Resonance Imaging
PEEK: Polyetheretherketone
PLA: Polylactic Acid
PSI: Patient-Specific Implant
RMSE: Root-Mean-Square Error
ROI: Region of Interest
STL: Standard Tessellation Language
Ti: Titanium
